# Biodiesel production from candlenut oil using a non-catalytic supercritical methanol transesterification process: optimization, kinetics, and thermodynamic studies

**DOI:** 10.1039/d2ra00571a

**Published:** 2022-03-29

**Authors:** Marwan Abdulhakim Shaah, Md Sohrab Hossain, Faisal Allafi, Mohd Omar Ab Kadir, Mardiana Idayu Ahmad

**Affiliations:** Environmental Technology Division, School of Industrial Technology, Universiti Sains Malaysia 11800 Penang Malaysia sohrab@usm.my mardianaidayu@usm.my +6046533678 +6046532216 +6046532214; Pultex Sdn Bhd Jalan Kampung Jawa, Bayan Baru 11950 Bayan Lepas Penang Malaysia

## Abstract

The present study was conducted to determine the feasibility of biodiesel production from candlenut oil using supercritical methanol (scMeOH) as a non-catalytic transesterification process. The influence of the scMeOH transesterification process was determined with varying pressure (85–145 bar), temperature (260–300 °C), methanol to oil (M : O) ratio (15 : 1–35 : 1), and reaction time (15–25 min). The experimental conditions of the scMeOH transesterification process were designed using central composite design (CCD) of experiments, and the process was optimized using response surface methodology (RSM). It was found that scMeOH temperature, pressure, M : O ratio, and reaction time substantially influenced the transesterification process. The maximum biodiesel yield of 96.35% was obtained at an optimized scMeOH transesterification process at the pressure of 115 bar, the temperature of 285 °C, M : O ratio of 30 : 1, and reaction time of 22 min. A second-order kinetics model and Eyring equations were utilized to determine the kinetics and thermodynamics of biodiesel production from candlenut oil. The activation energy value was determined to be 28.35 KJ mol^−1^. Analyses of the thermodynamic properties of biodiesel revealed that the transesterification process was non-spontaneous and endothermic. The physicochemical properties of produced candlenut biodiesel *via* scMeOH complied with most of the biodiesel properties as per ASTM D6751 and EN14214, thereby referring to good quality biodiesel production. The findings of the present study reveal that the scMeOH is an effective non-catalytic transesterification process for biodiesel production from candlenut oil.

## Abstract 2

The present study was conducted to determine the feasibility of biodiesel production from candlenut oil using supercritical methanol (scMeOH) as a non-catalytic transesterification process.

## Graphical abstract

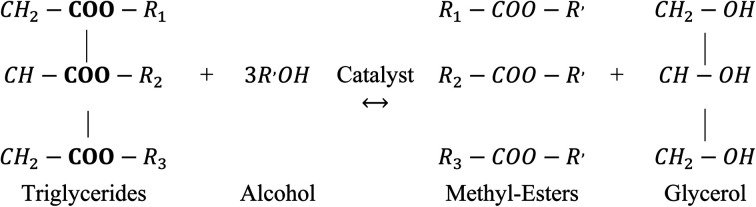

## 1. Introduction

Energy is one of the essential elements of our daily life, and it plays a prominent role in all commercial sectors, including transportation, power generation, agriculture, and various other industries.^1^ It has been reported that global energy consumption will increase rapidly by 50% in 2035 compared to that in 2012.^2,3^ Currently, fossil fuels such as coal, natural gas, and petroleum-derived products are the primary energy source. Nevertheless, these are finite energy sources and are rapidly depleting. One of the current global problems is the instability of petroleum production. Besides, there is increasing public concern worldwide to determine alternative energy sources to mitigate the growing demand for fossil fuels.^1,4^ Furthermore, many agreements have been made to reduce greenhouse gas emissions and develop new alternative energy sources to replace fossil sources.^5–7^

Biodiesel is a broad term used for the fuel produced from plant feedstock and biological sources such as vegetable plants, forestry by-products, waste, and animal fats.^8^ Generally, biodiesel is renewable, non-toxic, and it has a lower degradation time and lower emission rate than conventional petro-diesel.^9–12^ It has similar properties to petro-diesel, and it can be directly used in the existing engines without any need for engine modification.^11^ In the past, edible oils were the primary feedstock for biodiesel production.^12–14^ Several studies have been reported on the production of biodiesel from edible feedstocks such as canola, corn, sunflower, soybean, and palm oil.^12–15^ However, the use of edible oil as a biodiesel feedstock has some limitations, as it has a high production cost owing to the high prices of vegetable oils.^15^ Moreover, biodiesel production from edible oils has a significant influence on human food security, resulting in a reduction in food resources.^16^ To overcome the existing energy problems, more focus and emphasis have shifted towards non-edible oil crops as a feedstock for biodiesel.^16,17^ Numerous studies have focused on the production of biofuel from non-edible oils, including mahua (*Madhuca longifolia*), ratanjyot (*Jatropha curcas*), neem (*Azadirachta indica*), moringa (*Moringa oleifera*), and karanja (*Pongamia pinnata*).^14–18^ These feedstocks are available in abundance and do not affect the food chain.^9,18^

**Fig. 1:**
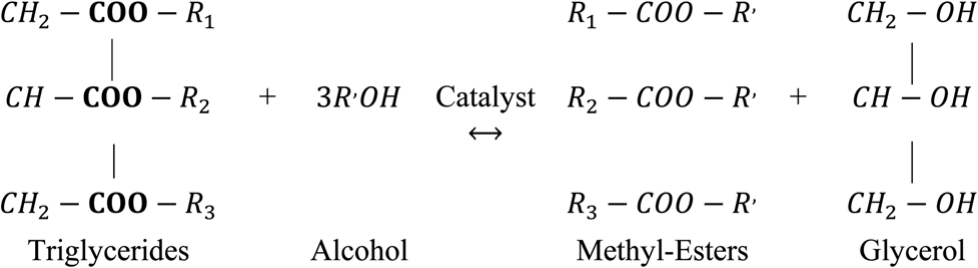
The basic catalytic transesterification reaction.

**Fig. 2:**
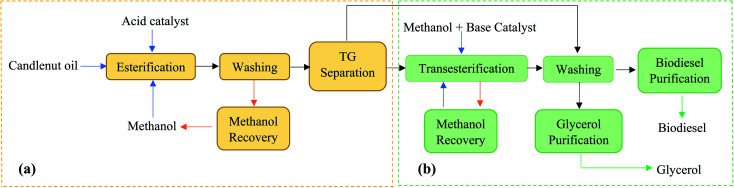
Biodiesel production pathway using two-step transesterification method: (a) esterification, (b) transesterification.

Among all the potential non-edible oils, candlenut is considered as a promising feedstock for biodiesel production, because of its high oil content and it can plant in non-fertile land like sandy, saline, and gravely soils that are not suitable for food production.^18–21^*Aleurites moluccana* L. Wild, known as candlenut or kemiri, is a flowering tropical tree belonging to the Euphorbiaceae family. Even though the candlenut tree is native to Indo-Malaysia, it is now one of the world's major domesticated multipurpose trees.^21^ It has a medium size with a crown shape, irregular branches with large green leaves, and a maximum length of 20 m. It grows rapidly in the semi-tropical and tropical climate under the following ideal conditions: 0–1200 m height, soils with pH 5–8, 18–30 °C temperature, and 600–4300 mm rainfall. Candlenut is also known as candleberry, Indian walnut, varnish tree, buah keras (Malaysia), or kukui nut (Hawaii).^21^ Its seed oil content varies from 50–60 wt%, and the average annual oil yield is 3000 kg ha^−1^.^20^ The extracted oil is used in paints, varnishes, and the production of high-quality biofuels.^19^ The main processes for synthesizing biodiesel are dilution, microemulsion formation, pyrolysis, and transesterification.^6,16^ Among these, catalytic transesterification is the most commonly used process for biodiesel production.^20,22^ The basic catalytic transesterification reaction is shown in Fig. 1. Two types of catalysts, homogeneous (acidic or basic) and heterogeneous (acidic, basic, or enzymatic), are used for the transesterification of vegetable oils.^13,23^ However, candlenut oil cannot be used in conventional base-transesterification methods without pre-treatment because of the high proportion of free fatty acids (FFAs). It has been reported that the FFA content of candlenut oil is more than 7%.^20^ To overcome this problem, a two-step reaction (acid catalytic esterification and base catalytic transesterification) was developed to reduce FFA content in the feedstock. In this process, the oil is pre-treated with an acid catalyst to reduce FFA content to less than 1%, followed by a base catalytic transesterification reaction to produce biodiesel. The biodiesel production pathway using the two-step transesterification method is shown in Fig. 2. The main disadvantages of the two-step process include the long reaction time and low recovery of the catalyst.^6,24–26^ In the literature, few studies have reported the use of a two-step transesterification technique for candlenut biodiesel production. Pham *et al.*^26^ produced candlenut biodiesel *via* a two-step transesterification method. Sulistyo *et al.*^27^ carried out a two-step transesterification of high FFA-containing candlenut oil to produce biodiesel.

**Fig. 3:**
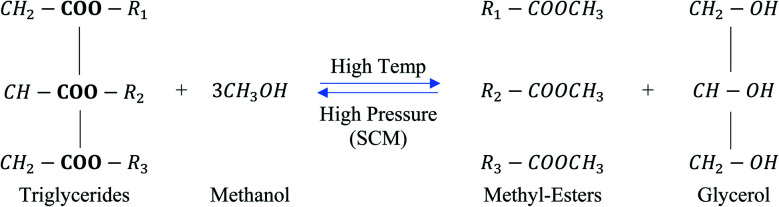
Supercritical methanol transesterification reaction of triglycerides.

Saka and Kusdiana^28^ developed the supercritical methanol (scMeOH) transesterification technology to overcome the problems associated with the catalytic transesterification method. This technology can produce biodiesel in a single step without the requirement of any catalyst. Biodiesel production from high FFA-containing feedstock and high biodiesel conversion are the main advantages of the scMeOH transesterification process.^28,29^ Moreover, the absence of a catalyst allows for simple purification and separation steps to be carried out during biodiesel production.^29^ Furthermore, as the temperature increases, the solubility of oil and methanol also increased, as the dielectric constant of the polar components decreases.^30^ The scMeOH transesterification of triglycerides is shown in Fig. 3. Supercritical methanol (scMeOH) transesterification reaction not only decreases the capital cost by leaving out soap separation and production of wastewater but is also able to produce high purity biodiesel from feedstocks with high FFA content.^30^ The separation of biodiesel is also easier since no catalyst is used, and the reaction can be completed in a shorter time compared to the two-step process.^28,31^ However, it should be noted that the process requires the elevation of pressure to a supercritical stage. The selected pressure should also be high enough to ensure solubility between the oil and methanol to enable high conversion efficiency in a short time frame.^31^

Although studies have been conducted on biodiesel production from candlenut oil, a knowledge gap exists in the optimization of candlenut biodiesel production method parameters that give the maximum biodiesel yield using RSM. The optimal biodiesel production parameters have never been investigated. In large-scale biodiesel production methods, it is very important to maximize the biodiesel yield at the lowest production cost. Optimizing each variable of the operational process would contribute to the improvement of the process and make it more efficient.^14,19^ This study aimed to produce biodiesel *via* a scMeOH transesterification process using candlenut oil. Additionally, the effects of temperature, pressure, time and methanol to oil (M : O) ratio on scMeOH were determined. The response surface methodology was used for the optimization of the scMeOH technology for biodiesel production. A kinetic and thermodynamic model was employed to analyze the scMeOH transesterification process. Finally, the physicochemical properties and fatty acid composition of the oil extracted from candlenut seeds and the resultant biodiesel were determined and compared with the ASTM and European standards. The findings of the present study will be applicable in producing biodiesel from non-edible oil containing high FFAs and moisture.

## 2. Material and methods

### 2.1 Sample collection and preparation

The candlenut seeds used in this study were collected from a local market in Penang Island, Malaysia. The seeds were dried at 104 °C for 4 h to remove the water content and then ground to the desired particle size for oil extraction. Candlenut oil was extracted using supercritical carbon dioxide technology. A 3 L supercritical reactor was loaded with 500 g of ground candlenut seeds, and the CO_2_ gas was delivered to the reactor using a high-pressure pump. The extraction was carried out at a pressure of 30 MPa and temperature of 60 °C for a duration of 90 min. Oil yield was measured using eqn (1) to calculate the oil content of candlenut seeds. Then, the physicochemical properties and fatty acid composition of the extracted candlenut oil were determined.1
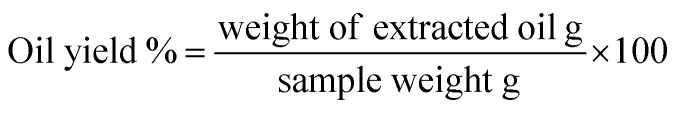


### 2.2 Transesterification of candlenut oil using scMeOH

**Fig. 4:**
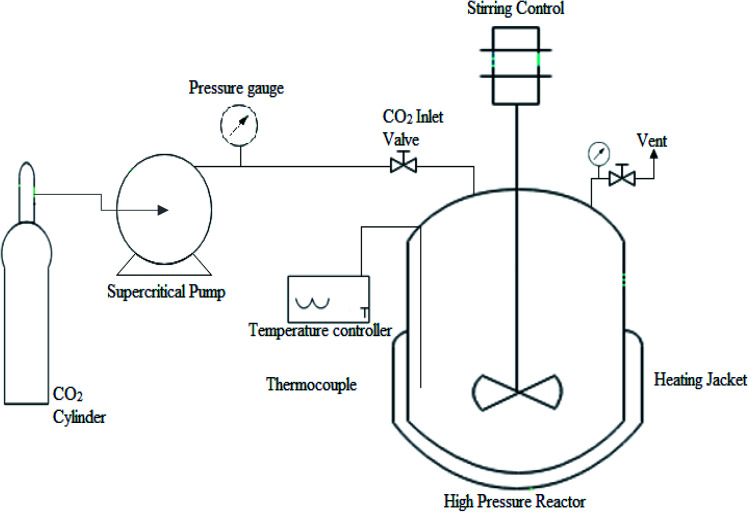
Experimental setup for the transesterification under supercritical methanol condition.

The experimental setup for transesterification of candlenut oil under scMeOH conditions is shown in Fig. 4. Crude candlenut oil was used for non-catalytic transesterification in a 300 ml modified autoclave batch reactor made of stainless steel (model 4520, PARR instrument, USA). A thermocouple was connected to a temperature controller to control the temperature in the reactor at a variance of error of less than 4 °C. Two pressure gauges, used to monitor the pressure, were placed next to the pump and inside the reactor. In a conical flask, candlenut oil and methanol were mixed at 60 °C for 10 min, before being placed in the vessel, and continuously stirred at 300 rpm. A high stirring speed above 500 rpm negligibly affected the ester content, as reported by Ryu *et al.*^32^ The pressure inside the vessel was maintained by purging with the N_2_ gas. After the target temperature was reached, CO_2_ was pumped into the reactor using a supercritical pump to increase the pressure inside the vessel to the target pressure. After achieving the desired conditions, the reaction was maintained for a specific period that had been set in advance. When the reaction was complete, the reactor was rapidly transferred to an ice bath to quench the reaction. The reactor was then opened, and the liquid content was transferred to a round flask for vacuum distillation. Later, the oil was weighed to determine biodiesel yield, which was calculated using the following equation:2
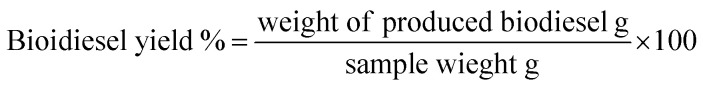


The CCD matrix was used for determining the conditions for the reaction experiment based on laboratory-located preliminary experiments. The process parameters that were chosen in this study were the reaction time (min), reaction temperature (^o^C), reaction pressure (bar), and M : O ratio. The variables and their levels were carefully selected following an examination of the parameters. Studies have indicated that increasing the temperature to more than 300 °C affects fatty acid composition. Thus, the maximum and minimum temperature ranges were chosen as 260 and 300 °C, respectively. Being a rapid method, the scMeOH reaction is normally completed within 15–25 min (observed in previous experiments). Thus, a minimum of 10 min is considered the shortest time required to complete the transesterification reaction under scMeOH conditions. Given this, the longer duration of the reaction (35 min) indicates that the efficiency of conversion is low. It has been suggested that the most likely cause for a low efficiency of conversion is the decomposition of esters over a longer reaction period. Therefore, the experiment reaction times were maintained between 10 and 30 min. Furthermore, the larger increase in temperature when scMeOH transesterification occurred could be impacted by the high M : O ratio. Hence, in practical terms, it is not cost-effective to use a substantial volume of methanol. Accordingly, it is preferable to reduce the amount of methanol as much as possible to minimize downstream separation and purification expenditure. Therefore, the M : O proportion was screened from the lowest ratio of 15 : 1 to the highest ratio of 35 : 1. The pressure directly affects the scMeOH transesterification reaction. To ensure that the working conditions in the reactor were above the critical pressure of methanol (above 80 bar), and since temperature is directly related to pressure, the reaction pressure was optimized. In summary, the reaction parameters selected were as follows: reaction time of 10, 15, 20,25, and 30 min; reaction temperatures of 260, 270, 280, 290, and 300 °C; M : O ratios of 15 : 1, 20 : 1, 25 : 1, 30 : 1, and 35 : 1; and reaction pressures of 85, 100, 115, 130, and 145 bar.

### 2.3 Experimental design

The experimental design (DOE) used RSM to optimize the reaction parameters for a greater yield of biodiesel. By utilizing RSM according to four factors and five levels of CCD, an investigation was conducted on the impact of four independent variables and their interactivity on the outcomes of the reaction (yield of biodiesel). RSM using the CCD method is the most popular among all optimization tools for reaction conditions. Full or fractional designs with center points are included; these integrate with a set of axial points, thereby allowing curvatures in the modeled results to be more successfully predicted. This study involved a range of independent variables examined through five levels. Coding for these was as follows: −*α*, −1, 0, +1, and +*α*, as shown in Table 1. The CCD design was observed, resulting in a 4-factor and 5-level design being introduced. A total of 30 experiments were performed, as shown in Table 2. Calculations for the total number of experiments were performed according to eqn (3).

3 *T* = 2*n* + 2*n* + *m*

where *n* refers to the number of independent variables, and *m* refers to the number of replicated center-points. Four independent variables were included in this study; hence, sufficient details should be derived, assisting the second-order polynomial models to be predicted for the responses and the yields of biodiesel and glycerol. A total of 30 experiments were devised based on 16 factorial points and eight axial points. The performance of the experiments was random and included six replicated versions at the center point to allow precise prediction of experimental errors. All runs of the experiment were carried out in a randomized manner in order to keep the impact of any unexplained, inconsistent aspect of the responses to a minimum.^33^ The reaction variables under analysis were the M : O molar ratio (*A*), temperature (*B*, ^o^C), pressure (*C*, bar), and time (*D*, min).

**Table 1:** Experimental design variables and their coded levels

Symbol	factor	Unit	Levels				
			−*α*	−1	0	+1	+*α*
*A*	Methanol to oil ratio	M : O (%)	15	20	25	30	35
*B*	Reaction temperature	^o^C	260	270	280	290	300
*C*	Reaction pressure	Bar	85	100	115	130	145
*D*	Reaction time	Min	10	15	20	25	30

**Table 2:** Experimental design based on RSM for supercritical methanol transesterification of candlenut biodiesel

Run No.	Coded process variables				Biodiesel yield%	
	*A*	*B*	*C*	*D*	Actual	Predicted
1	0	0	0	0	89.60	89.58
2	0	0	−*α*	0	82.55	82.32
3	0	*α*	0	0	93.55	93.83
4	1	1	−1	1	93.90	94.18
5	0	0	0	0	89.20	89.58
6	1	1	1	−1	86.60	87.028
7	−1	−1	1	−1	77.20	76.93
8	1	−1	−1	1	83.90	84.37
9	0	0	0	0	89.70	89.58
10	0	0	α	0	90.50	91.01
11	−*α*	0	0	0	73.60	74.22
12	α	0	0	0	81.15	80.82
13	1	1	−1	−1	81.90	81.44
14	−1	1	1	1	93.90	93.67
15	0	0	0	α	96.10	95.99
16	0	0	0	0	89.30	89.58
17	−1	1	−1	−1	78.45	78.54
18	1	−1	1	−1	80.15	79.83
19	0	0	0	0	89.75	89.58
20	1	1	1	1	96.90	96.68
21	−1	1	1	−1	85.60	84.81
22	1	−1	1	1	86.90	86.79
23	−1	−1	−1	−1	70.850	70.75
24	−1	−1	−1	1	80.40	79.98
25	−1	1	−1	1	90.50	90.47
26	−1	−1	1	1	82.95	83.09
27	0	−*α*	0	0	76.15	76.15
28	0	0	0	0	89.94	89.58
29	1	−1	−1	−1	74.10	74.34
30	0	0	0	−*α*	76.75	77.11

### 2.4 Statistical analysis

A quadratic polynomial regression model was developed to describe the relationships between the reaction parameters and repetitions and to enable predictions of biodiesel yields as functions of the independent variables. The general quadratic polynomial eqn (4) is given as follows:4
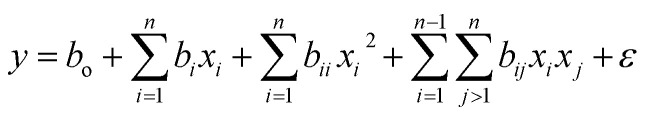
where *y* is the response (yield of biodiesel), *b*_o_ is the intercept value, *b*_*i*,_*b*_*ii*,_ and *b*_*ij*_ are the linear, quadratic, and interactive coefficients, respectively, and *x*_*i*_ and *x*_*j*_ are independent variables (*j* ≠ *i*). *n* refers to the number of independent variables, and *ε* is the random error. Design Expert Software Version 7.0.0 (Stat-Ease, Inc. 2021 East Hennepin Ave., Suite 480 Minneapolis, MN 55413) was used to perform all statistical analyses.

### 2.5 Kinetic and thermodynamic model of biodiesel production

Although the second-order rate constant is commonly used for adsorption studies, the second-order rate equation has also been employed to describe the kinetic behavior of liquid–liquid conversion.^25^ The present study used a second-order rate model to describe the kinetic behavior of biodiesel production from candlenut oil using scMeOH transesterification. The production of biodiesel from candlenut oil can be expressed as follows:5
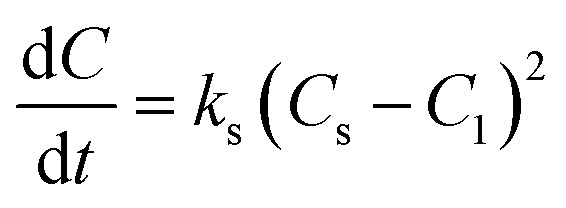
where *k*_s_ is the second-order rate constant (g mg^−1^ min^−1^), *C*_*t*_ is the weight of biodiesel upon scMeOH transesterification at a given time *t* (mg g^−1^), and *C*_s_ is the equilibrium concentration of the biodiesel produced *via* scMeOH (mg g^−1^). Eqn (5) can be integrated at the initial and boundary conditions (*t* = 0 to *t*, and *C* = 0 to C) and can be rearranged as follows:6
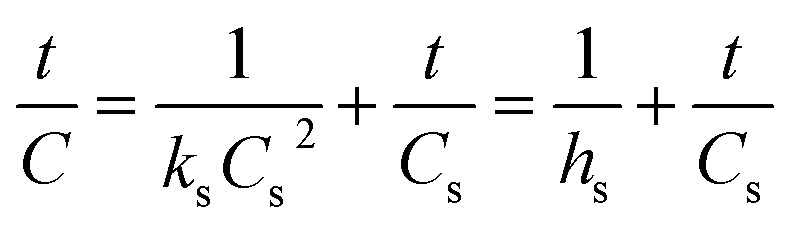
where *h*_s_ (mg g^−1^ min^−1^) is the initial transesterification rate at time *t* = 0. The activation energy (*E*_a_, kJ mol^−1^) was estimated for biodiesel production from candlenut oil using the Arrhenius law, as shown in eqn (7):7
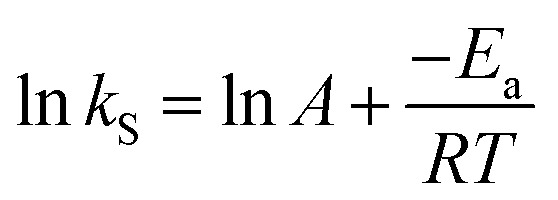
where *A* is the frequency factor (g mg^−1^ min^−1^), *T* is the absolute temperature (K), and *R* is the gas constant (8.31 J mol^−1^-K). The entropy (Δ*S*_a_, kJ mol^−1^ K^−1^) and enthalpy (Δ*H*_a_ kJ mol^−1^) for biodiesel production from candlenut oil were determined using the Eyring theory, as shown in eqn (8).8
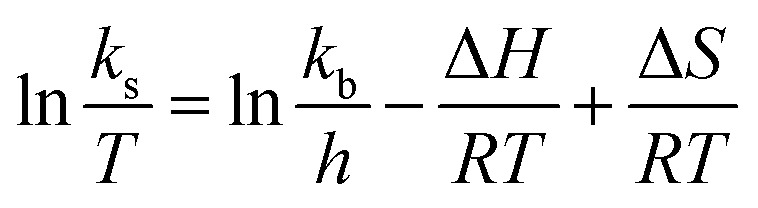
where *K*_b_ is the Boltzmann constant (1.38 × 10^−23^ J K^−1^), and *h* is the Planck constant (6.63 × 10^−34^ J s^−1^). The Gibbs free energy (Δ*G*, kJ mol^−1^) for biodiesel production from candlenut oil was determined using the following equation:

9 Δ*G* = Δ*H* − *T*Δ*S*

### 2.6 Physicochemical properties of candlenut lipids and biodiesel

The viscosities of the extracted candlenut oil and biodiesel were determined using a viscometer. The densities of oil and biodiesel were determined following the procedure described by the ASTM D1298 for density measurements. The saponification values of candlenut oil and biodiesel were determined following the AOAC920.160 standard test method. Briefly, approximately 1 g of the sample was mixed with 25 ml of 0.5 N alcoholic KOH, followed by titration with 0.5 HCl. The acid values of both candlenut oil and biodiesel were determined using the AOAC 940.28 standard method. A phenolphthalein indicator (1 ml) was used to quantify the oil and biodiesel titrated using a 0.1 N KOH solution. The acid values of candlenut oil and biodiesel were estimated using the following equation:10
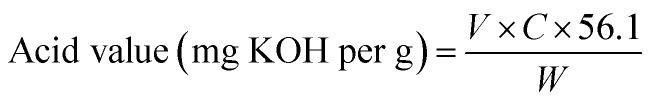
where *C* is KOH solution concentration (mol V^−1^), *V* is KOH volume (ml), and *w* is the weight of the sample (g). Using the obtained value, the FFA contents of the oil and biodiesel were calculated using eqn (11).11
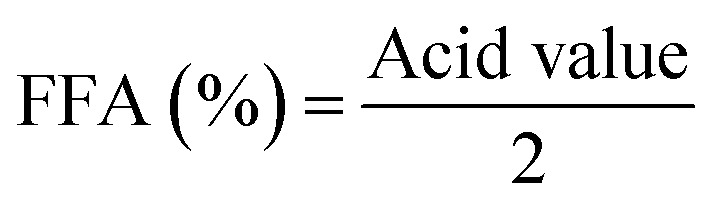


The iodine numbers of candlenut oil and Biodiesel were determined using the AOAC 920.159 test method. The peroxide values of the samples were determined following the standard AOAC 965.33 method. The cloud point (CP) and pour point (PP) were determined using the jar test method according to the steps described in ASTM D2500 and ASTM D7683, respectively. The moisture content of lipids and biodiesel was determined following the AOAC 930.15 test method. The calorific values of both lipids and biodiesel were measured using the bomb calorimeter method following the ASTM D5865 standard using an Isoperibol Calorimeter (6200, Parr Instruments, USA). Approximately 0.05 g of liquid samples was placed in a crucible, and the bomb was filled with oxygen gas (O_2_) up to 25 atm. The test was started after placing the crucible in the calorimeter. The calorific value was recorded after burning was completed. The cetane number was determined following the ASTM D613 test method.

### 2.7 GC-FID and FTIR analyses

The fatty acid compositions of lipids and biodiesel were determined *via* gas chromatography (GC), and the fatty acids were analyzed by converting them to their respective fatty acid methyl esters (FAMEs). Approximately, 0.1 μg of biodiesel was injected into the fused silica capillary column (30 m × 0.25 mm, id, 0.25 μm). The temperature of the detector and injector was 250 °C, and pure helium was used as the carrier gas. Initially, the temperature of the oven was kept at 100 °C for 10 min, before being raised at 10 °C per min until a final temperature of 240 °C was achieved, which was held for 15 min. A standard FAME mixture (Supelco 37-component FAME Mix, Sigma Chemicals, USA) was used to estimate fatty acids. The percentage of fatty acids in the lipids and biodiesel was determined using an internal normalization technique.

The characteristics of the bBiodiesel from candlenut were determined using a Fourier transform infrared (FTIR) spectrometer (PerkinElmer System 2000 FTIR), which comprised a detection device with a spectral range of 4000–600 cm^−1^ with a resolution of 4 cm^−1^. FTIR spectra were analyzed using the Nicolet OMNIC 5.01 software provided with the apparatus. The fatty acid esters present in the biodiesel from candlenut were identified from FTIR spectrum absorption bands.

## 3. Results and discussion

### 3.1 scMeOH transesterification of candlenut oil

**Fig. 5:**
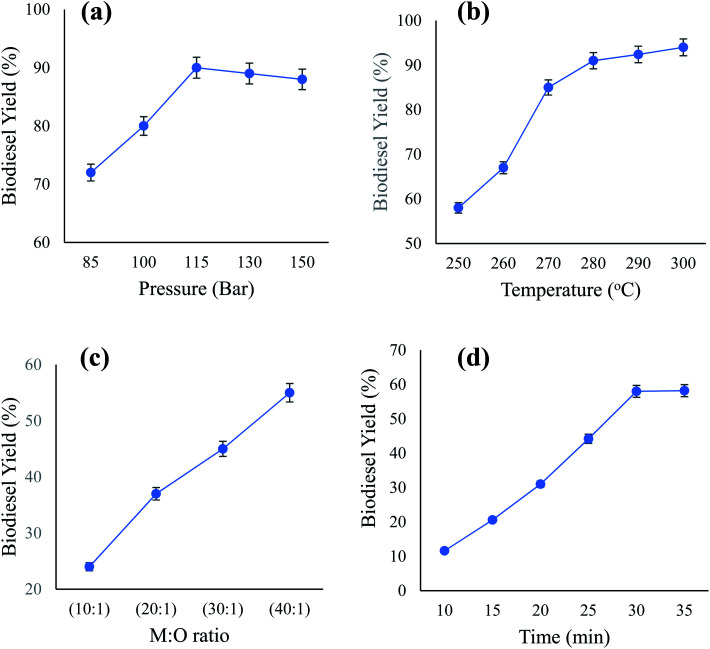
Production of biodiesel from candlenut oil by scMeOH transesterification, (a) effect of pressure, (b). Effect of temperature, (c) effect of methanol to oil ratio, and (d) effect of reaction time.

The effect of scMeOH on the production of biodiesel from candlenut oil was determined at various temperatures (250–310 °C), pressures (85–150 bar), and M : O ratios (10 : 1–40 : 1). The reaction time (10–35 min) is shown in Fig. 5. It was found that the scMeOH pressure, temperature, ratio, and conversion time substantially influenced biodiesel production from candlenut oil. The percentage of biodiesel increased with an increase in pressure from 85 to 115 bar (Fig. 5(a)). However, the amount of biodiesel produced was negligible with an excessive increase in pressure to 150 bar. The highest biodiesel yield obtained was 90% at a pressure of 115 bar, temperature of 250 °C, M : O ratio of 30 : 1, and conversion time of 30 min. The reaction speed increased with increasing pressure, as higher levels of methanol solubility and molecular activity were derived from reactions wherein the pressure was increased. This explains the increase in the rate of the reaction and the relative likelihood of particle collision. When the reaction pressure increases, it allows greater splitting of the triglyceride and methyl combination bonds and becomes FAME.

The biodiesel production from candlenut oil using scMeOH transesterification increased with an increase in temperature from 250 °C to 280 °C at a pressure of 85 bar, M : O ratio of 30 : 1, and reaction time of 30 min (Fig. 5(b)). The amount of biodiesel increased gradually with a further increase in temperature from 280 °C to 310 °C. Approximately 55% of biodiesel was produced at a temperature of 250 °C; the percentage of biodiesel production increased to 91.4% at a temperature of 290 °C and reached a maximum of 92.6% at 300 °C. Similarly, Rahimi *et al.*^34^ also found that an increase in temperature increases biodiesel yield, and the highest yield is obtained at 290 °C. Their study demonstrated that at a higher temperature, biodiesel yield starts to decrease, as the extremely high temperature leads to the degradation of methyl esters. Furthermore, Ghoreishi and Moein^35^ reported that an excessive increase in temperature above 300 °C causes the decomposition of FAMEs and results in a reduction in biodiesel yield. Fig. 5(c) shows that the amount of biodiesel increased with an increase in the M : O ratio. In the transesterification process, methanol is responsible for moving the equilibrium toward the product (biodiesel), and the subsequent maximum possible conversion occurs using extra methanol. The higher M : O molar ratios resulted in a transesterification process with greater efficiency because the contact of methanol with triglycerides increased. An increase in the M : O ratio from 10 : 1 to 20 : 1 increased biodiesel yield, and the maximum biodiesel yield was 55% at a M : O ratio of 40 : 1, pressure of 85 bar, temperature of 250 °C, and reaction time of 30 min. However, biodiesel yield decreased at a higher M : O ratio (40 : 1). Aboelazayem *et al.*^6^ reported similar results for the transesterification of cooking oil at an M : O ratio of 37 : 1; Ghoreishi and Moein^35^ reported that 33 : 1 is the optimum M : O ratio for producing biodiesel from waste vegetable oil. Furthermore, the findings of this study also agree with the results reported by Santana *et al.*,^36^ who demonstrated that a high M : O ratio leads to a reduction in biodiesel production. At higher levels, excess methanol interferes with the separation of glycerin due to increased solubility. Fig. 5(d) shows that the amount of biodiesel increased with an increase in reaction time from 10 to 20 min at a pressure of 85 bar, M : O ratio of 30 : 1, and temperature of 250 °C. The highest, about 91.4% of lipids, was separated at a separation time of 30 min, pressure 85 MPa, and temperature of 290 °C.

### 3.2 Development of a regression model

The results obtained from all the variables (M : O, *T*^o^C, *P*_bar_, and *T*_min_) employed in this study are summarized in Table 3. The software fitted the linear, quadratic, and cubic outcomes from the factors of the experiment onto a model, and the effects of the interactions among them were also fitted. The yield was predicted using the model as a function of the given factors. The statistical model was chosen according to various statistical assessments, including the adjusted coefficient of determination (*R*_adj_^2^), predicted coefficient of determination (*R*_pre_^2^), lack of fit, and associated aliased coefficients. Eqn (12) represent the developed quadratic models with empirical relationships between responses and reaction variables within specific levels in terms of experimental factors.

12 *y*_BD_ = 89.58 + 1.65*A* + 4.42*B* + 2.17*C* + 4.72*D* − 0.17*AB* − 0.17*AC* + 0.20*AD* + 0.023*BC* + 0.68*BD* − 0.77*CD* − 3.02*A*^2^ − 1.15*B*^2^ − 0.73*C*^2^ − 0.76*D*^2^

where *y*_BD_ is the yield of biodiesel. Meanwhile, *A*, *B*, *C*, and *D* refer to the experimental variables, which include the M : O ratio, heat level, pressure, and duration. The impact of each reaction variable on the response is illustrated by regression equations. A synergetic impact is indicated by the positive sign of each term, whereas a negative sign indicates an antagonistic effect.^6,32^ The impact of the reaction variable on the response is represented by a linear coefficient. Meanwhile, the interactive effect on the response of the process variables is represented by the coefficient of variable interaction. Finally, the impact on the response of variable excess is represented by a quadratic coefficient.

**Table 3:** Analysis of variance for biodiesel and glycerol yield for the developed model

Source	Sum of square	DF	Mean square	*F* value	*P* value
	Biodiesel	Biodiesel	Biodiesel	Biodiesel	Biodiesel
Model	1467.13	14	104.79	477.58	<0.0001
*A*	65.34	1	65.34	297.77	<0.0001
*B*	469.05	1	469.05	2137.60	<0.0001
*C*	113.10	1	113.10	515.43	<0.0001
*D*	535.05	1	535.05	2438.42	<0.0001
*AB*	0.46	1	0.46	2.13	0.1643
*AC*	0.47	1	0.47	2.16	0.1614
*AD*	0.64	1	0.64	2.91	0.1083
*BC*	0.00	1	0.00	0.03	0.8502
*BD*	7.29	1	7.29	33.22	<0.0001
*CD*	9.45	1	9.45	43.09	<0.0001
*A* ^2^	249.36	1	249.36	1136.43	<0.0001
*B* ^2^	36.05	1	36.05	164.29	<0.0001
*C* ^2^	14.52	1	14.52	66.19	<0.0001
*D* ^2^	15.69	1	15.69	71.52	<0.0001
Residual	3.29	15	0.21		
Lack of fit	2.89	10	0.28	3.65	0.0827
Pure error	0.39	5	0.07		
Cor total	1470.42	29			

As eqn (6) shows, all linear coefficients have positive signs, indicating that biodiesel yield increases when any one process variable (O ratio, *T*^o^C, *P*_bar_, and *T*_min_) increases. Eqn (6) and (7) evidently show that varying factor A, the M : O ratio, produced the most substantial impact on the yields of biodiesel and glycerol, since, amongst the variables, its coefficient is the largest. Furthermore, the adequacy of the model in the prediction has been assessed; hence, all errors linked to normality assumptions can be reported. Biodiesel yield ranged from 70.85–96.9%, depending on the conditions of each experiment. As all the linear terms and quadratic terms, except the interaction terms *AB*, *AC*, *AD*, and *BC*, were found to be significant model terms in maximizing the biodiesel yield, the adequacy of the models in the predictions was checked using several analyses. The predicted model accuracy was evaluated using the coefficient of correlation (*R*^2^). The closer the *R*^2^ approaches unity, the better the indication of similarity between the actual and predicted values. The *R*^2^, *R*_pre_^2^, and *R*_adj_^2^ values for the biodiesel predicted model were 0.9978, 0.9883, and 0.9985, respectively. These results indicate that almost 99.7% of the sum of the variation is qualified by the variables in the experiment for biodiesel yield.

**Fig. 6:**
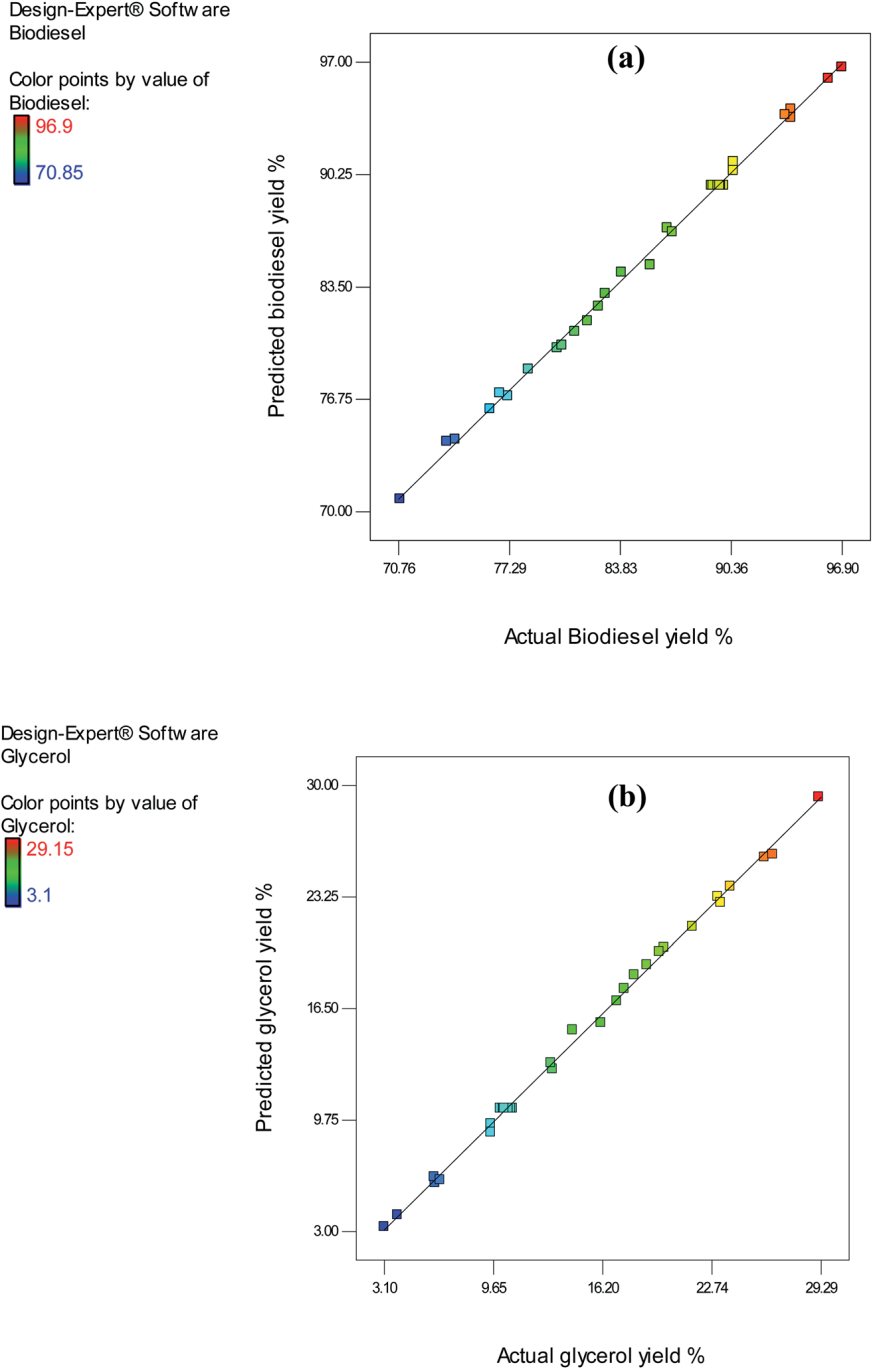
Predicted *versus* actual values for biodiesel model (a) and glycerol yield (b).

The significance of the predicted models was determined using statistical data obtained from variance analysis. Furthermore, the interactions between the reaction parameters and the significance of their impacts were examined. ANOVA produced the parameter values for the yield of biodiesel as presented in Table 3. The model of biodiesel was assessed for their significance according to *p*-values and *F*-tests at a confidence level of 95%. The lesser the *p*-value than 0.05, the greater is the significance of the correlated parameter. Our observations showed the high significance of both models for biodiesel, where the *p*-value was <0.0001. Such measurements ensure that the model is significant in representing the outcomes of real experiments. Additionally, one type of ANOVA analysis, lack of fit, measures the extent to which the model failed in its representation of the data points in the experiment. A non-significant value for lack of fit indicates a model with a high fit. The biodiesel lack fit value was 0.0827. The test's non-significance illustrates the successful representation of the data from the experiment in the models. Furthermore, the similar nature of the predicted and actual data ensures that the model accurately predicts the response variables. Fig. 6(a) and (b) illustrate the actual and predicted data from the experiment, using a model created to depict the yield of biodiesel.

**Fig. 7:**
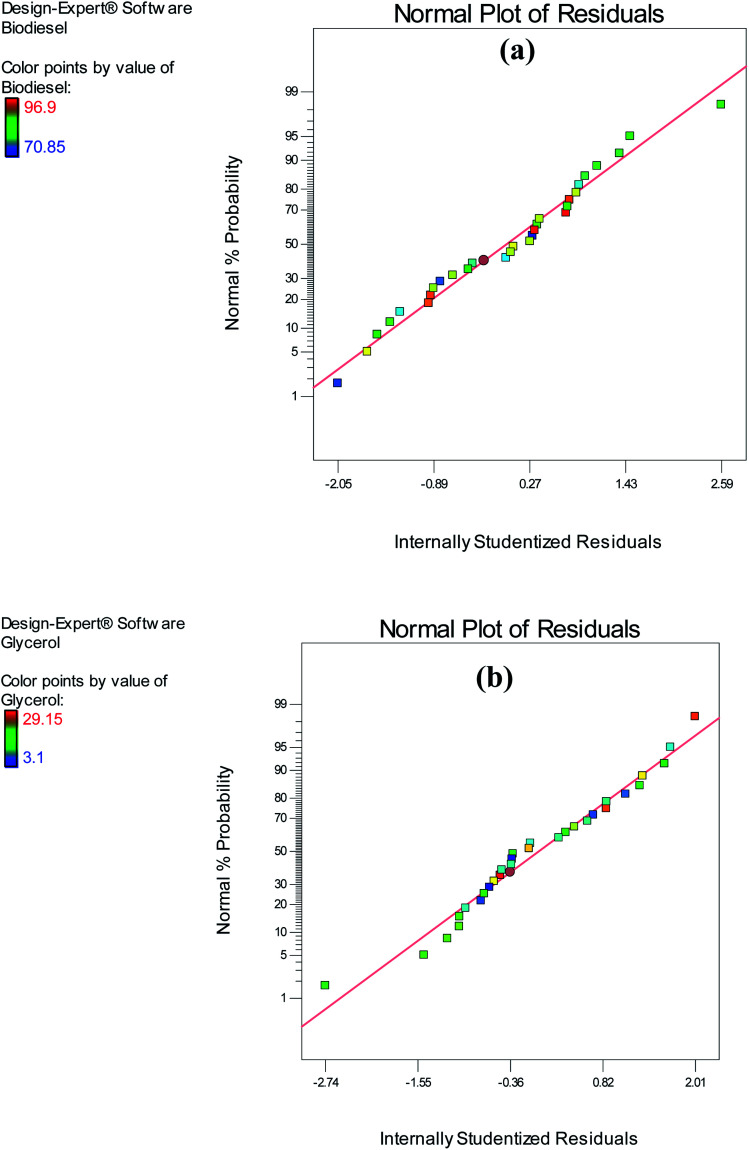
Normal plot of residuals for (a) biodiesel yield model and (b) glycerol yield model.

The significance of each parameter was determined using ANOVA. As shown in Table 3, all the examined factors had a significant linear impact on the yield of biodiesel. The *p*-values for the M : O ratio (*A*), temperature (*B*), pressure (*C*), and reaction time (*D*) were less than 0.05, for biodiesel. Furthermore, in each quadratic model, the corresponding coefficients were positive for all the factors associated with biodiesel yield. As shown in the analyses, the interaction between the variables temperature – duration (*BD*) and pressure – duration (*CD*) had a significant impact on the yield of biodiesel. Conversely, the interactivity of the variables with other factors had a non-significant impact on the yield of biodiesel. Furthermore, verifying the assumptions of ANOVA was vital because it was used in the predicted model validation.^6^ An investigation of the normality of residuals was conducted utilizing a normal plot, and relatively straight lines were formed, as shown in Fig. 7(a) and (b). The validity of the first assumption was ensured by this test, as a normal distribution of residuals was obtained for the model of biodiesel.

### 3.3 Effects of process variables and their interactions

The impact of individual parameters and their interactions was studied to analyze and investigate the influence of parameter variations on the responses.

#### 3.3.1 Effect of single variable on the response

**Fig. 8:**
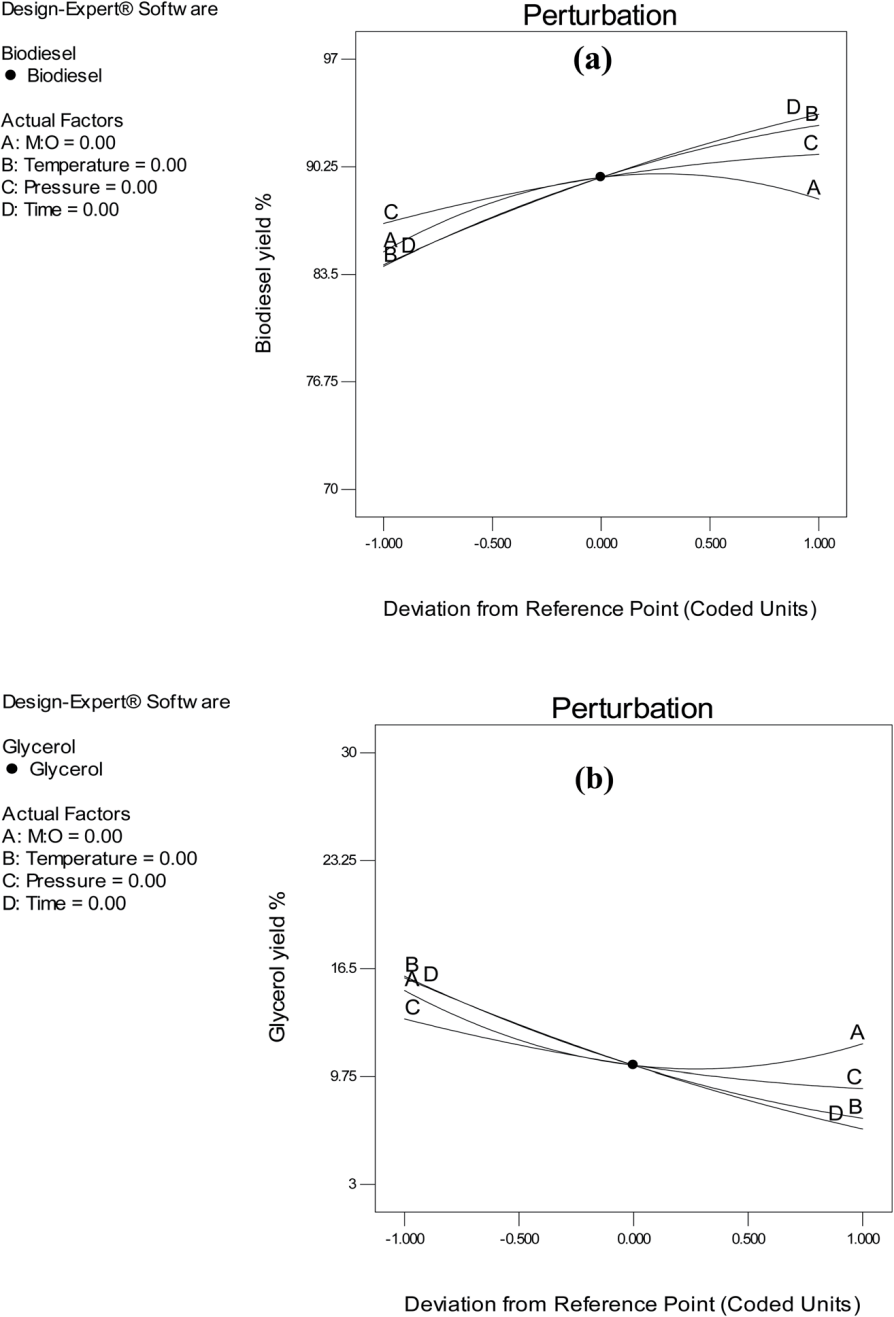
Perturbation plot showing the effect of individual variables on (a) biodiesel yield and (b) glycerol yield.

The impact of the reaction variables at specific spatial points can be compared using perturbation plots. For this study, the center point of each variable was selected to provide a consistent point for comparison among all variables. The pattern in which the yields of biodiesel was influenced by specific reaction variables is shown in Fig. 8(a). A key drawback to the use of scMeOH in biodiesel production is that a high volume of methanol is required. Hence, it is important to investigate the effect of scMeOH on biodiesel yield by considering how to optimize the process. An increase in the M : O ratio results in a reduction in biodiesel yield. This negative impact is shown in Fig. 8(a). A previous study by Aboelazayem *et al.*^6^ on high fatty acid waste cooking oil, where he reported a negative influence of the M : O ratio on biodiesel yield, agrees with the present findings. According to Nan *et al.*,^37^ increasing the M : O ratio during biofuel synthesis using non-catalytic scMeOH has a significant impact on biodiesel yield. The authors provided an explanation for their results, suggesting that the homogenous reaction phase required only reduced molar ratios. Accordingly, an increased M : O ratio had no significant impact on solution homogeneity in the present study. Conversely, the M : O ratio positively affected the yield of glycerol. This outcome was anticipated, since previous reports suggested that the M : O ratio improves the transesterification reaction used to produce glycerol.^36^

The temperature of the reaction is the primary parameter when using scMeOH for biodiesel production. A high reaction temperature leads to the thermal degradation of biodiesel. According to Imahara *et al.*,^38^ the yield of biodiesel is negatively affected by reaction temperatures above 300 °C. Furthermore, the yield of biodiesel is negatively affected by low reaction temperatures. As the critical methanol temperature is 239 °C, the range of temperature selected in this study was 260–300 °C. Our findings showed that the yield of biodiesel was positively affected by the reaction temperature. As shown in Fig. 8(a), the yield of biodiesel increased with an increase in reaction temperature, which is in agreement with the results of a previous study, which reported that the yield of biodiesel is positively impacted by a rise in temperature.^37^ Conversely, lower reaction temperatures had a positive effect on the yield of glycerol.

Another important parameter in scMeOH transesterification is the reaction pressure. It has a substantial effect on solution properties, such as the intensity of hydrogen bonds and density.^6^ It has been reported that the effect of reaction pressure on biodiesel yield is not highly significant. Nevertheless, in this study, the reaction pressure had a significant effect on the biodiesel yield. As the supercritical pressure of methanol is 80 bar, the pressure range selected was 85–145 bar. The results of this study agree with those of Tsai *et al.*,^39^ who indicated that the yield of biodiesel varies by approximately 10% when the pressure is increased from 100 to 250 bar. Nonetheless, the yield of glycerol was not significantly affected by the reaction pressure, as illustrated in Fig. 8(b). Increasing the reaction pressure to 130 bar led to a reduction in glycerol yield, yet a drop in reaction pressure slightly affected the yield of glycerol. Furthermore, the shorter duration of the scMeOH transesterification reaction is one of its advantages over the catalyzed methods. In this study, the duration was selected as 15–30 min, as recommended in previous studies.^6^ Results showed that the reaction time had the greatest impact on the yield of biodiesel. Biodiesel yield increased with an increase in the reaction time from 15 to 25 min. Conversely, the reaction duration showed an inverse relationship with glycerol yield. An increase in the reaction time led to a reduction in glycerol yield.

#### 3.3.2 Effect of variables interactions on the response

**Fig. 9:**
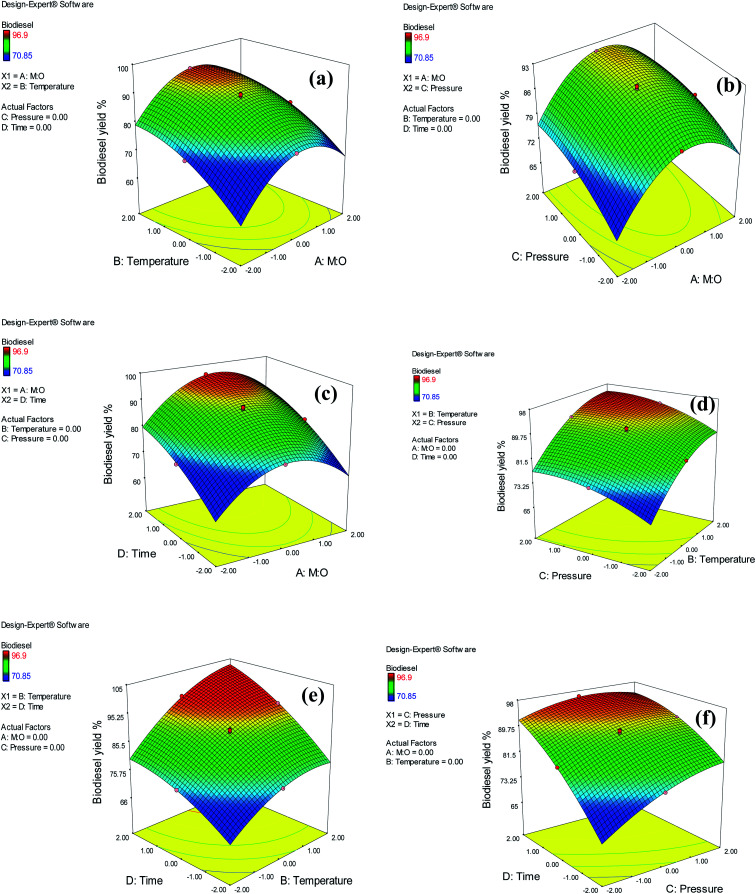
3D response surface plot of interaction effect between: (a) M : O and temperature, (b) M : O and pressure, (c) M : O and reaction time, (d) temperature and pressure, (e) temperature and reaction time, and (f) pressure and reaction timer on biodiesel yield.

The interaction plots and ANOVA results were used to observe the influence of interactions among each pair of variables on biodiesel yield. Furthermore, contour plots and 3D surface, for the yield of biodiesel compared to the way the two independent variables interacted, were utilized to demonstrate the influence of the interactions on the responses. In each plot, the two independent variables remained constant at their center point. To simplify the process, this analysis refers only to the biodiesel yield response. The results shown in Table 3 reveal that there are two terms of interaction that significantly affect the biodiesel yield, reaction temperature, reaction time (*BD*), reaction pressure, and reaction time (*CD*). The interactive results of the reaction temperature and reaction time had a significant impact on biodiesel yield. The 3D response surface plots for temperature and reaction duration on the biodiesel yield are illustrated in Fig. 9(e). This graph shows that when the temperature is low, the reaction time has a very low positive effect, whereby higher temperatures of a reaction have a positive impact on the yield of biodiesel. Moreover, with a shorter reaction time, the rising temperature of the reaction showed a slightly positive effect on the yield of biodiesel. However, with longer reaction times, biodiesel yield increased with an increase in temperature. On the other hand, an increase in access temperature resulted in a reduction in biodiesel yield. These findings illustrate the importance of studying the interactive effects of variables. Shin *et al.*^40^ examined the yield of biodiesel from highly acidic rapeseed oil using scMeOH transesterification. Their results suggest a reduced impact on biodiesel yield at reaction temperatures over 300 °C at a constant M : O ratio of 40 : 1. Their explanation for this is the increase in the rate of thermal degradation of methyl esters.

The interactions between the reaction pressure and reaction time had a significant impact on the yield of biodiesel. The pressure in the reactor was increased by pressurizing the CO_2_ gas, which was used as a co-solvent to raise the reaction pressure to the desired pressure using a supercritical pump. Furthermore, CO_2_ enhances the solubility of methanol in oil. The 3D response surface plot for the reaction pressure and time for biodiesel yield is shown in Fig. 9(f). As assessed from ANOVA (Table 3), there was a significant interaction effect between reaction pressure and time, which is also confirmed by the graph in Fig. 9(f). The reaction pressure had a negligible effect on biodiesel yield at shorter reaction periods. However, a slightly negative effect of the reaction pressure was observed at longer reaction durations. Ong *et al.*^41^ reported that the impact of increasing pressure is not crucial, as it exceeds the critical pressure of methanol. They explained that both transesterification and esterification involve the same number of moles of reactants and products. Hence, a change in pressure does not affect the chemical equilibrium of the reaction, according to Le Chatelier's principle. The negative effect of an increasing pressure might result from FAME degradation, as CO_2_ reduces the critical point of the system and hence requires a milder temperature.^6^ The 3D response surface plots of the insignificant effects of the interactions between (a) M : O and temperature, (b) M : O and pressure, (c) M : O and reaction time, (d) temperature and pressure, and (e) temperature and reaction time are shown in Fig. 9.

### 3.4 Process optimization and experimental validation

Optimization of the reaction variables affecting biodiesel yield from high fatty acid candlenut oil using RSM has not been studied before. To optimize the reaction responses (the yield of biodiesel), the optimal conditions for achieving the desired objective were evaluated by implementing the numerical features of the Design expert 7.0.0 software. Subsequently, the software-generated 30 solutions for optimal conditions, from which the most desirable solution was selected. The resulting optimum conditions resulted in 96.35% yields for biodiesel at a 30 : 1 M : O molar ratio, 285 °C, and 115 bar pressure in 22 min of reaction time. To validate the predicted optimum conditions, three experiments were conducted under these conditions, and the average result was considered as the experimental outcome. The experimental validation resulted in a biodiesel yield of 96.13%, which demonstrated the adequacy of the predicted optimum conditions within a relative error of 0.22% from the experimental results. The candlenut biodiesel yield and the optimal experimental conditions of scMeOH transesterification were almost similar, as reported by Singh *et al.*^31^ The study reported that the maximum biodiesel production of 95.67% from jojoba oil using the supercritical methanol transesterification process at the optimum conditions of reaction temperature 287 °C, reaction pressure of 123 bar, methanol to oil ratio of 30 : 1, and reaction time of 23 min.^31^

### 3.5 Kinetic and thermodynamic modeling

**Fig. 10:**
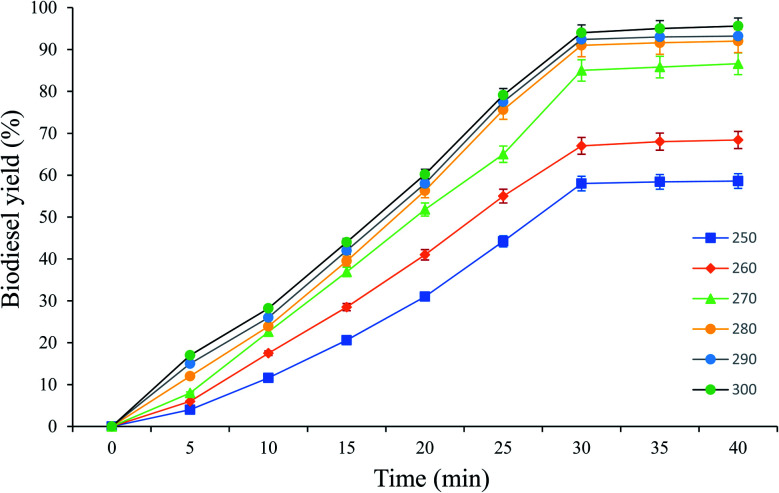
Biodiesel production from candlenut oil using scMeOH transesterification reaction at pressure 110 bar with varying temperature and reaction time.

**Fig. 11:**
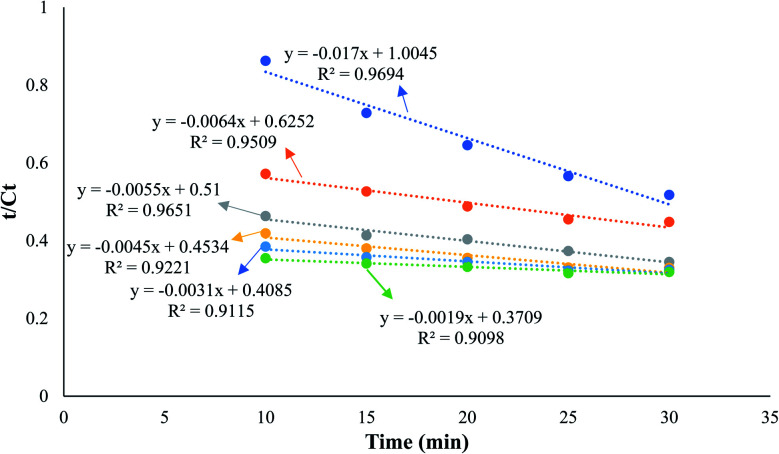
Second-order kinetic model for the biodiesel production from candlenut oil using scMeOH transesterification reaction with varying temperatures, ranging from 250 to 300 °C.

As shown in Fig. 5(a), the optimal scMeOH pressure was 110 MPa at a temperature of 250 °C, an M : O ratio of 30 : 1, and a reaction time of 25 min to produce biodiesel from candlenut oil. Fig. 10 shows biodiesel production from candlenut oil at 110 bar with varying temperatures (250–300 °C) and reaction times (5–30 min). It was found that biodiesel yield increased with increasing temperature and reaction time and reached equilibrium at 30 min conversion time. Accordingly, kinetic and thermodynamic studies were conducted for biodiesel production from candlenut oil *via* the scMeOH reaction at 110 bar and varying temperatures (250–300 °C) and reaction time (5–30 min). A second-order kinetic model equation was used to express the overall conversion curves of biodiesel from candlenut oil at 110 bar, as shown in Fig. 11. Based on the second-order rate model shown in eqn (5) and (6), the estimated correlation results are listed in Table 4. It was found that the *h*_s_, *k*_s_, and *C*_s_ values significantly increased with an increase in temperature from 250 °C to 300 °C. This result suggests that rapid biodiesel production from candlenut oil can be achieved using the scMeOH reaction with the parameters reported in the current study.^42,43^ Furthermore, the high correlation coefficient (*R*^2^), greater than 0.90, indicates that the proposed second-order kinetics model adequately describes the scMeOH conversion behavior of biodiesel from candlenut oil at temperatures in the range of 250–300 °C.^43^

**Table 4:** The second-order kinetic model and thermodynamic parameters for the production of biodiesel from candlenut oil using scMeOH transesterification reaction

Temperature (°C)	Second-order kinetic model			*R* ^2^	Thermodynamic quantities of activation		
	*h*_s_ (mg g^−1^ min^−1^)	*k*_s_ (mg mg^−1^ min^−1^)	*C*_s_ (mg g^−1^)		Δ*H* (kJ mol^−1^)	Δ*S* (J mol^−1^ K^−1^)	Δ*G* (kJ mol^−1^)
250	0.995	3.44	58.82	0.9694	231.22	26.57	164.79
260	1.59	39.05	156.25	0.9509			162.13
270	2.105	69.61	181.81	0.9651			159.48
280	2.205	108.91	222.22	0.9221			156.82
290	2.44	254.73	322.58	0.9115			154.16
300	2.69	746.85	526.31	0.9098			151.51

**Fig. 12:**
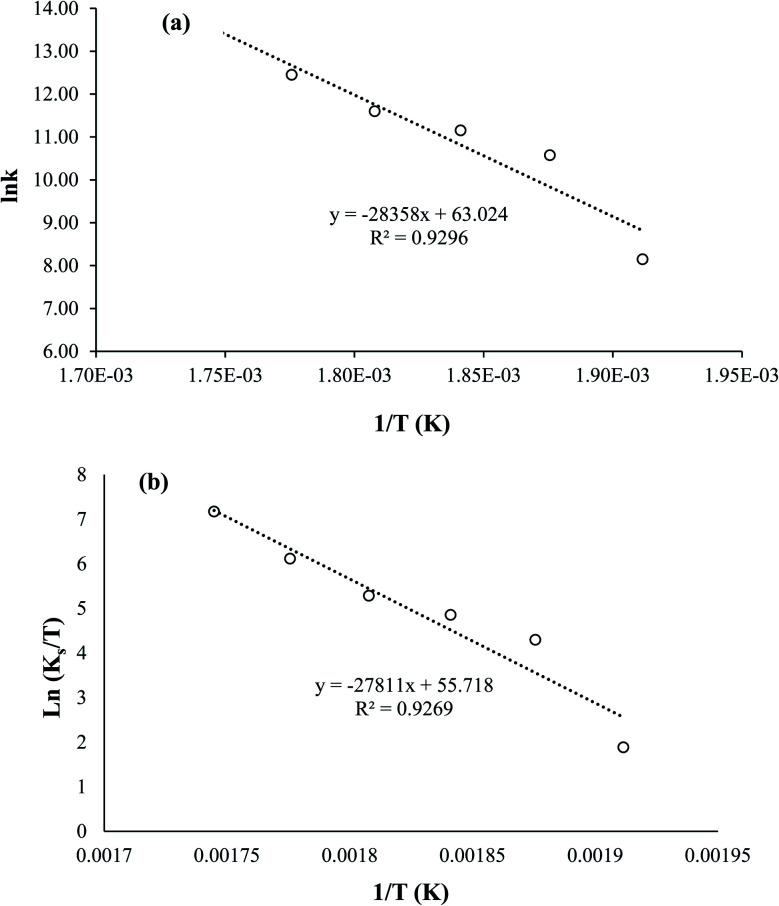
Relationships between the absolute temperature (1/*T*) and the second-order rate constant, (a) ln(*k*) and (b) ln(*k*/*T*) for the biodiesel production from candlenut oil using scMeOH transesterification reaction.

Fig. 5 shows the relationship between the absolute temperature and second-order rate constant for estimating the activation energy (Fig. 12(a)) and thermodynamic properties (Fig. 12(b)) for the production of biodiesel from candlenut oil. The estimated activation energy for biodiesel production from candlenut oil using the scMeOH reaction was 28.35 kJ mol^−1^. The positive activation energy reveals that the scMeOH transesterification process is a temperature-dependent process, indicating the production of biodiesel increased with increasing temperature.^38^ The kinetics behavior of the scMeOH transesterification process varies with the supercritical CO_2_ (ref. 42) extraction process. For instance, Mohammad Ilias *et al.* [2022] observed that the lipids extraction from chicken by-product waste using scCO_2_ was not temperature-dependent. However, the estimated activation energy value for the scMeOH transesterification process was lower than other non-catalytic transesterification processes like the microwave-assisted transesterification process.^43^ The estimated Δ*H* and Δ*S* values were 231.22 kJ mol^−1^ and 26.57 kJ mol^−1^ K^−1^, respectively. The positive Δ*H* value indicated that biodiesel production from candlenut oil was endothermic. The Δ*G* of activation decreased with an increase in temperature. The estimated positive Δ*G* values revealed that biodiesel production from candlenut oil using the scMeOH reaction was unspontaneous. Moreover, the high correlation coefficient (0.9269) for estimating the thermodynamic properties of activation reveals that the transition state theory reliably describes the biodiesel production from candlenut oi using scMeOH reaction between 250 °C and 300 °C.

### 3.6 Physicochemical properties of candlenut biodiesel

In the present study, oil was extracted from candlenut seeds using the scCO_2_ extraction method. Approximately 63% of the oil was separated from candlenut seeds at a pressure of 35 MPa, a temperature of 60 °C, and an extraction time of 90 min. The extracted oil was used as a feedstock for biodiesel production using the scMeOH transesterification reaction. The physicochemical properties of the separated candlenut oil and the biodiesel produced from candlenut oil, using optimized experimental conditions of scMeOH transesterification (30 : 1 M : O, 285 °C, 125 bar, and 22 min), were analyzed and compared with a biodiesel standard, and other biodiesel feedstocks as shown in Table 5. Viscosity is an important parameter of biodiesel because it affects the fuel injection system, causes poor fuel combustion, and increases exhaust emissions. Here, the viscosity decreased with an increase in temperature. The viscosities of the extracted candlenut oil and biodiesel were found to be approximately 25.80 and 4.8 mm^2^ s^−1^, respectively. The viscosity of candlenut oil was found to be lower than that of wild radish oil (36.32 mm^2^ s^−1^) and higher than that of Jatropha oil, (19.77 mm^2^ s^−1^).^20,43,44^ However, the viscosity of candlenut biodiesel was found to be lower than the biodiesel produced from Jatropha, jojoba and castor as they were 4.84 mm^2^ s^−1^, 15.4 mm^2^ s^−1^, and 5.86 mm^2^ s^−1^, respectively. Lower viscosity will improve the fuel flow through the ignition system, which resulting in a good fuel combustion and lower the exhaust emission. Moreover, the viscosity of candlenut biodiesel was found to comply with the biodiesel international standards (EN14214 and ASTM D 6751). Density is one of the important properties of biodiesel fuel; it has a direct effect on the engine performance because the motor fuel injection system operates on a volume metering system. The densities of candlenut oil and biodiesel were found to be approximately 914 and 871 kg m^−3^, respectively. The density of the extracted candlenut oil was slightly lower than the densities of jatropha oil and radish oil (918 and 918.8 kg m^−3^).^20^ The candlenut biodiesel has a lower density than Jatropha biodiesel and castor biodiesel which are 879 kg m^−3^, and 946 kg m^−3^, while the density of castor biodiesel was the same as the density of candlenut biodiesel. Furthermore, the density of the synthesized biodiesel was found to comply with the biodiesel standard limits (800–900 kg m^−3^) according to the EN14214 standard.

**Table 5:** Properties of extracted candlenut lipids and candlenut biodiesel

Property	scCO_2_ extracted candlenut oil	Candlenut biodiesel	Jatropha biodiesel^4^	Caster biodiesel^46^	Jojoba biodiesel^47^	ASTM 6751	EN14214
Density (kg m^−3^) @ 15 °C	914 ± 5	871 ± 6	879	946	871	—	860–900
Viscosity (mm^2^ s^−1^)	25.8 ± 3	4.8 ± 2	4.84	15.4	5.86	1.9–6	3.5–5
Pour point (^o^C)	2 ± 0.08	−2.3 ± 0.1	3	−30	−6	−15 to 16	—
Cloud point (^o^C)	10 ± 1	3 ± 1	2.8	−18	−2	−3 to 12	—
Moisture content (%)	0.1 ± 0.01	0.01 ± 0.01	0.02	0.04	0.053	>0.03	>0.05
Iodine number gI_2_/100 g	122 ± 2	130 ± 2	—	78.21	74.7	—	120 max
Cetane number	—	51 ± 1	51	43.7	—	>47	51
Acid value (mg KOH per g) oil	15.8 ± 1.2	0.38 ± 0.02	0.3	2.8	0.22	0.5 max	0.5 max
FFAs (%)	7.9 ± 0.6	0.19 ± 0.01	0.15	1.4	0.11		
Saponification value (mg KOH per g)	172 ± 2	187 ± 1.5	—				
Peroxide value (meq kg^−1^)	8.6 ± 0.8	5.8 ± 0.5	—				
Calorific value (MJ kg^−1^)	42 ± 2	42.3 ± 1.2	38.5	38.3	42.8	—	35

The acid value and FFAs are critical parameters for biodiesel production.^44^ The quality of biodiesel improves with a reduction in the AC and FFA content.^20^ The estimated acid value and FFAs for the extracted candlenut oil were 15.8 mg KOH per g and 7.9%, respectively. In the two-step transesterification process for biodiesel production, the FFAs must be first subjected to an esterification process to reduce the FFA content to less than 1% to facilitate the occurrence of transesterification reaction and biodiesel production. However, the scMeOH transesterification method produces biodiesel without the need for a pre-treatment step (esterification).^29^ The acid value and FFAs of the produced biodiesel were found to be 0.38 mg KOH per g and 0.19%, respectively, which are similar to the Jatropha biodiesel and slightly higher than the acid value of jojoba biodiesel and castor biodiesel. The acid value of the produced candlenut biodiesel is desirable because it complies with the biodiesel standard ranges described in ASTM D6751 and EN 14214.

The presence of peroxides in oil and biodiesel indicates its rancidity and oxidation stability. Research has shown that the desired peroxide value for essential oils is less than 10 meq kg^−1^, and the rancidity of biodiesel over the peroxide value is 50 meq kg^−1^.^39,43^ The peroxide values in candlenut lipids and the produced biodiesel were 8.6 and 5.8 meq kg^−1^, respectively. Thus, it can be presumed that the oxidation stability of the biodiesel produced from candlenut oil is good. The saponification number indicates the presence of fatty acid chains in the oil [Sohrab]. The saponification values of candlenut oil and biodiesel were 172 and 187 meg KOH per g oil. The saponification value of the produced biodiesel was below the saponification value limit described in the ASTM D6751 standard. The iodine number is related to the degree of unsaturation and the stability of oils and biodiesel. It measures the number of double bonds that react with iodine, resulting in the polymerization of fuel due to epoxide formation *via* the addition of oxygen in double bonds. As the iodine number increases, the number of unsaturated fatty acids increases, which lowers the stability of biodiesel. In this study, the iodine number values of candlenut oil and biodiesel were 122 and 130 I_2_/100 g, respectively. The iodine number of candlenut biodiesel was found higher than the iodine value of jojoba and castor biodiesel, which is due to the presence of the unsaturated fatty acids in candlenut biodiesel feedstock. However, the iodine number of the produced biodiesel was found to be above the biodiesel standard limit.

The cetane number is another important property of biodiesel, as it has a direct effect on fuel ignition. A better ignition quality of the fuel is always associated with a higher cetane value. The cetane number affects several engine performance parameters such as drivability, combustion, white smoke, stability, CO and HC emissions, and noise.^40^ The cetane number of candlenut biodiesel was 51, which was higher than the castor biodiesel and similar to Jatropha biodiesel. It was found that the cetane number of the produced biodiesel complied with the EN14214 standard limit.

The CP and PP were used to measure fuel usability at cold temperatures. The higher CP and PP values indicate poor cloud flow properties of biodiesel. The CP and PP values increase with an increase in the levels of saturated compounds in the biofuel;^39,44^ in this study, the CP and PP values for biodiesel were found to be within the ASTM D6751 standard range. The calorific value indicates the energy content of the oil and biofuel. The calorific value has a direct effect on the engine performance in terms of torque and maximum horsepower. A higher calorific value results in high temperatures and improves engine performance during the combustion of biodiesel. The calorific values of candlenut oil and biodiesel were 42 and 42.3 MJ kg^−1^, respectively. The calorific value of the produced biodiesel was found to be slightly lower than that of the biodiesel produced from jojoba oil (42.82 MJ kg^−1^) and higher than the calorific values of jatropha oil, caster oil, and jojoba oil, which were 38.5 MJ kg^−1^, 38.34 MJ kg^−1^, and 38.33 MJ kg^−1^, respectively.^20,41^ The calorific value of candlenut biodiesel complied with the EN14214 standard range. These findings indicated that candlenut oil can be used as a potential feedstock for biodiesel production.

As shown in Table 5, the candlenut biodiesel properties comply with both international standards (ASTM D6751 and EN14214) which makes it a good biodiesel fuel that can be used directly in the existing engines. Moreover, comparing the main properties of the candlenut biodiesel with other biodiesel like Jatropha biodiesel, jojoba biodiesel, and castor biodiesel showed that the candlenut biodiesel has better values with the biodiesel standard range. The viscosity and density of candlenut biodiesel were found lower than other biodiesel, which is more desirable for biodiesel fuel to improve fuel combustion, avoid engine knocking and decrease exhaust emission. Furthermore, the cetane number and the calorific value of candlenut biodiesel were found higher than other biodiesels and within the international biodiesel standards. FAME composition of the scCO_2_-extracted candlenut oil and biodiesel was determined using GC-FID analyses, as shown in Table 6. It was found that candlenut oil contains 10.98% saturated fatty acids, 24.7% mono-unsaturated fatty acids, and 64.20% polyunsaturated fatty acids. Linoleic acid (C18:2) was the most abundant (40.1%), followed by oleic acid (C18:1) (24.7%) and linolenic acid (C18:3) (24.1%). Furthermore, candlenut biodiesel showed a higher proportion (89.92%) of the unsaturated fatty acid methyl esters. The degree of unsaturation of fatty acid methyl esters significantly affects the cold flow properties and oxidation stability.^45^ Thus, candlenut biodiesel is presumed to have low oxidation stability.

**Table 6:** FAME composition of extracted candlenut oil and biodiesel^a^

Fatty acid	Carbon number	Fatty acid groups	Fatty acid (%)	
			Oil	Biodiesel
Caprylic acid	C8:0	SFA	0.04	0
Capric acid	C10:0	SFA	0.07	0
Lauric acid	C12:0	SFA	0.19	0.07
Myristic acid	C14:0	SFA	0.3	0.1
Palmitic acid	C16:0	SFA	6.9	6.6
Stearic acid	C18:0	SFA	2.9	3.6
Oleic acid	C18:1	MUFA	24.7	27.2
Linoleic acid	C18:2	PUFA	40.1	40.4
Linolenic acid	C18:3	PUFA	24.1	23.2
Arachidic acid	C20:0	SFA	0.58	0.5
ΣSFA			10.98	10.87
ΣMUFA			24.7	27.2
ΣPUFA			64.20	63.60

a SFA: saturated fatty acids, MUFA: mono-unsaturated fatty acids, PUFA: poly-unsaturated fatty acids.

**Fig. 13:**
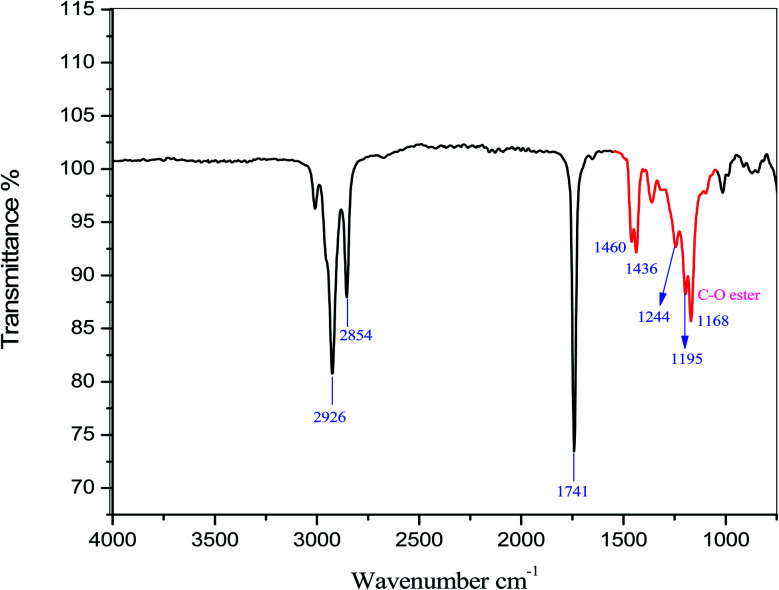
Fourier transform infrared spectrum for the candlenut biodiesel.

Fig. 13 shows the FTIR spectra of candlenut biodiesel. It can be clearly seen that the biodiesel derived from candlenut comprises long-chain fatty acid esters. In the range of 1800–1700 cm^−1^, the strong peak observed at 1741 cm-1 is assigned to C=O, which typically belongs to esters. The peaks located at 2926 and 2854 cm^−1^ confirmed the presence of =C–H asymmetric stretching vibration and –CH_2_ symmetric stretching vibrations, respectively, in candlenut biodiesel. The major spectral region for biodiesel is in the range of 1500–1000 cm^−1^. The peaks located at 1460 and 1436 cm^−1^ indicate bending vibrations of –CH_2_, and the high point located at 1244 cm^−1^ indicates the bending vibrations of –CH_3_. The peaks located at 1244, 1195, and 1168 cm^−1^ indicate the C–O–C anti-symmetric stretching vibration.

## 4. Conclusion

The present study utilized the scMeoH transesterification process to produce biodiesel from candlenut oil. It was observed that pressure, temperature, M : O ratio, and reaction time of the scMeOH significantly influenced the transesterification process for the biodiesel production. The maximum biodiesel yield obtained was 96.35% at the optimized scMeOH transesterification process, such as the pressure of 115 bar; temperature of 285 °C; M : O ratio of 30 : 1; and reaction time of 22 min. From the kinetics studies, the activation energy was determined to be 28.35 kJ mol^−1^, indicating the scMeoH transesterification process was temperature dependent. The thermodynamics properties analyses showed that Δ*H*, Δ*S*, and Δ*G* values were 231.22 kJ mol^−1^, 26.57 J mol^−1^ K^−1^, and 164.79–151.51 kJ mol^−1^,respectively. The estimated thermodynamic property values indicate that the biodiesel production from the candlenut oil using the scMeOH as a non-catalytic transesterification process was endothermic and non-spontaneous. The determination of fatty acids properties in the isolated biodiesel showed that saturated and unsaturated fatty acids content were 10.87% and 63.60%, respectively. The physicochemical properties analyses of the biodiesel showed that density, acid value, cloud point, pour point, saponification values, and iodine values were complied with the international biodiesel standards of ASTM D6751 and EN14214. The findings of the present study indicated that the scMeOH is an effective non-catalytic transesterification process for the biodiesel production from candlenut oil. Therefore, the scMeOH is transesterification process could be applied as an effective alternative of the conventional catalytic transesterification process for the sustainable production of biodiesel from non-edible oil.

## Conflicts of interest

There are no conflicts to declare.

## Supplementary Material
